# Development of Paper Sludge Ash-Based Geopolymer and Application to Treatment of Hazardous Water Contaminated with Radioisotopes

**DOI:** 10.3390/ma9080633

**Published:** 2016-07-28

**Authors:** Zhuguo Li, Toshihiko Ohnuki, Ko Ikeda

**Affiliations:** 1 Graduate School of Science and Technology for Innovation, Yamaguchi University, 2-16-1 Tokiwadai, Ube 755-8611, Japan; li@yamaguchi-u.ac.jp; 2 Advanced Science Research Center, Japan Atomic Energy Agency, 2-4 Shirakawa Shirane, Tokai-Mura 319-1195, Japan; ohnuki.toshihiko@jaea.go.jp

**Keywords:** geopolymer, paper sludge ash, radioisotope, radioactively contaminated water

## Abstract

Ambient temperature geopolymerization of paper sludge ashes (PS-ashes) discharged from paper mills was studied by means of scanning electron microscopy (SEM), X-ray diffraction (XRD), induction coupled plasma atomic emission spectrometry (ICP-AES), and X-ray absorption near edge structure (XANES). Two varieties of alkaline liquors were used in the PS-ash based geopolymers, corresponding to aqueous Na-metasilicate and Na-disilicate compositions. PS-ashes were found to be semi-crystalline and to have porous structures that make it possible to absorb much liquor. Flexural strengths of PS-ash-based geopolymers with liquor/filler ratios (L/F) of 1.0–1.5 ranged from 0.82 to 1.51 MPa at 4 weeks age, depending on PS-ashes and liquors used. The reaction process of the constituent minerals of the PS-ash is discussed. Furthermore, we attempted to solidify hazardous water contaminated with radioisotopes. Non-radioactive strontium and cesium nitrates were added as surrogates at a dosage of 1% into the PS-ash-based geopolymers. Generally, high immobilization ratios up to 99.89% and 98.77% were achieved for Sr^2+^ and Cs^+^, respectively, depending on the source of PS-ashes, alkaline liquors, and material ages. However, in some cases, poor immobilization ratios were encountered, and we further discussed the causes of the instability of derived geopolymer gels on the basis of XANES spectra.

## 1. Introduction

A geopolymerization technique may involve three categories of application. The first is the synthesis of silicates of nanometer scale at temperatures as low as 800 °C with less alkali contamination [1,2,3,4]. The second is the preparation of monolithic materials, potentially replacing Portland cement and recycling various sorts of wastes or by-products [5,6,7,8,9,10,11,12,13,14,15,16,17,18]. The third is the immobilization of hazardous heavy metals or radioisotopes contaminated in sludgy solid wastes, an application dating back to 1988 [19]. On the other hand, there is a liquid type of waste contaminated with radioisotopes awaiting to be treated [20,21]. To immobilize this type of waste by geopolymerization, the liquor/filler ratio (L/F) becomes large. However, if the geopolymer is made from general aluminosilicate powder (e.g., metakaolin and fly ash), a higher L/F would cause bleeding, and hazardous elements accordingly escape from the geopolymer. Although this problem would be solved by using coal fly ash cenospheres or porous glass ceramics [20,21], collecting or producing these materials in large quantities is very difficult and costly. Therefore, most studies are focused on the solid–liquid separation techniques based on zeolite media’s absorption [22,23,24]. Incidentally, this kind of zeolite absorption technique is practically limited to Cs.

On the other hand, according to literature [25,26], the annual discharge of paper sludge amounts to 3.1–3.8 million tons in Japan as dry-base. To reduce the volume as well as the bad odor, incineration is generally carried out at temperatures 600 °C–1000 °C, employing fluidized bed boilers. Accordingly, a huge amount of incineration ash (so-called paper sludge ash, PS-ash in short in the present study) is discharged. Some studies have attempted the recycling of PS-ash; for instance, hydrothermal solidification to produce building materials, solidification materials of soft ground, coagulants for dredging, etc. [27,28,29]. The PS-ash with high kaolin content has a pozzolanic characteristic [30]. However, beneficial use of the PS-ash on a massive scale is still one of the urgent environmental issues in Japan, as well as globally.

Several years ago, the authors found that the PS-ash can be used to produce geopolymers as active filler, and the geopolymers must have a L/F over 1.0 due to the high water absorption ability of PS-ash. This characteristic makes PS-ash-based geopolymer to be suitable for the disposal of radioactively-contaminated water [31,32]. In the present study, the reaction process of PS-ash-based geopolymer is studied from the aspects of mineralogy. Moreover, in order to clarify the stability of immobilization of radioisotopes, the immobilization ratios of radioisotopes in the PS-ash-based geopolymer is further investigated as a function of material age.

## 2. Experimental Procedure

### 2.1. Specimen Preparation and Strength Test

Three kinds of PS-ash were collected from paper mills in Japan. Their physicochemical characteristics are shown in Table 1. Two kinds of geopolymer liquors were prepared, designated as #0 and #1 liquors, respectively. The #1 liquor was prepared from condensed commercial Na-disilicate liquor (Na_2_O·2SiO_2_·aq) by diluting with deionized water to have a specific gravity (S.G.) of 1.27, corresponding to a 24.25% concentration of Na-disilicate (Na_2_O·2SiO_2_). The #0 liquor was prepared by mixing the #1 liquor with 10 M NaOH solution at a ratio of 3:1 by volume to have a S.G of 1.31, corresponding to a 23.55% concentration of Na-metasilicate (Na_2_O·SiO_2_).

Plain geopolymer pastes were prepared as tabulated in Table 2 without adding non-radioactive strontium and cesium sources. One hundred grams of each PS-ash was weighed and transferred to a 500 mL plastic beaker, and was hand-mixed with the alkaline liquors to obtain pastes. Then, the charge was cast into a three-cell type prismatic mold with 20 mm × 20 mm× 80 mm dimensions and was cured in a 20 °C, 100% RH chamber overnight. After demolding, the prismatic test pieces were kept curing for 28 days under the same conditions. Then, flexural strength was measured by employing a three-point flexural tester under the conditions of 50 mm span and 0.2 mm/min cross-head speed. Mean fracture load of three test pieces was used to calculate the flexural strength, applying the well-known three-point formula [31]. Bulk densities of the specimens were also measured before the flexural test.

On the other hand, paste specimens were also prepared, containing non-radioactive Sr-nitrate, Sr(NO_3_)_2_, and Cs-nitrate, CsNO_3_, together as surrogates. The surrogates and the solidified bodies are called SC and SC-GP in short, respectively. The dosage of each surrogate was 1% as nitrates against PS-ash weight. After weighing, the charge was bottle-mixed. The other procedures and conditions are the same as for the plain specimens.

### 2.2. Various Analysis of PS-Ashes and Leaching Test of Surrogates

The PS-ashes as received were chemically analyzed by X-ray fluorescence (XRF), employing Philips MagixPro (Royal Philips, Amsterdam, Holland) with fundamental parameter methods (FP). Loss on ignition (LOI) was separately determined by heating with an electric furnace at 1000 °C for 2 h. Furthermore, moisture content was measured with an oven at 105 °C for 2 h. Apparent densities were determined by a pycnometer. Then, specific surface areas were measured by a conventional Blaine apparatus (Marubishi Kagaku, Tokyo, Japan). Constituent minerals were identified by X-ray diffractometry (XRD), employing Rigaku RINT-2550 (Rigaku, Tokyo, Japan). To avoid reflections from glass, window-type sample holders made of Al metal were used throughout this measurement. Scanning electron microscopy (SEM) was adopted to observe textures of the PS-ashes, employing Jeol JSM-7600F (Jeol, Tokyo, Japan). XRD technique was also used to analyze the hardened paste specimens.

After 4 weeks aging, the SC-GP and plain specimens were further cured in ambient air for 2 weeks (4 + 2 week age), 8 weeks (12 week age), and 20 weeks (24 week age), respectively, in order to examine the stability of immobilization of the surrogates. After curing, the SC-GP samples were weighted and then crushed to be finer than 4 mm in size for the leaching test. Twelve point five grams of the crushed sample was immersed into the commercially available buffer solution (pH 4.01) containing phthalic salt, meant to mimic exposure to strong acidic rainfall at disposal sites. There are several methods to evaluate dissolutions as described in literature [31], but the phthalic salt buffer method is adopted as a laboratory scale method for the convenience of our laboratory facilities. The weight of the buffer solution was 125 g, 10 times the sample weight. Then, leaching test was performed in a 250 mL plastic bottle rotated at 60 rpm for 6 h. Finally, the leachates collected by percolating with filter paper were chemically analyzed by induction coupled plasma atomic emission spectrometry (ICP-AES), employing PerkinElmer, Optima 8300 (PerkinElmer, Waltham, MA, USA). Obtained concentrations of leachates were recalculated on the basis of 125 g to get leaching weights of the surrogates, while surrogate amounts were calculated to 12.5 g of SC-GP samples on the basis of filler/Na-silicates/water proportions and the resultant bulk densities of each age shown in Table 2 and Table 4, respectively. Details are as follows:

(1) Bulk densities obtained were regarded as apparent densities of the sample grains with the size of under 4 mm (Table 4).
(2) Density (D_0_) at 4 week age was disintegrated into filler/Na-silicates/water ratios according to the percentages shown in Table 2. For instance, for series 0-3SC shown in Table 4, D_0_ = 1.49, 1.49 was revolved into 0.677/0.191/0.625. Incidentally, the 4 week curing was performed in air (20 °C, 100% RH) so that the proportions in Table 2 are maintained in the solidified specimens.
(3) Then, the density (D_1_) at 4 + 2 week age was recalculated, supposing only moisture water was evaporated during the air dry. For instance, for 0-3SC, D_1_ = 1.08, which becomes 0.677/0.191/0.211.
(4) Moreover, the proportions of D_1_ in (3) were converted into percentages. For instance, for 0-3SC, D_1_ becomes 62.74/17.70/19.56, in total 100%.
(5) Finally, the filler amounts in dissolution test samples were obtained. For instance, for 0-3SC, the filler amount is 62.74%. Therefore, in the 12.5 g samples, the surrogate amount become 78.4 mg for strontium and cesium as nitrates, respectively, since the dosages were 1% of fillers by mass, irrespective of surrogate species (Table 6).
(6) The procedure is the same for other prolonged age specimens of 12 and 24 week ages of which bulk densities are indicated as D_2_ and D_3_, respectively (Table 4).

Thus, immobilization ratios were obtained as follows:

Immobilization ratio (%) = (1 − Sr^2+^ or Cs^+^ in 125 g leachate/Sr^2+^ or Cs^+^ in 12.5 g sample) × 100 (1)

In present study, the surrogates were regarded as a part of alkaline liquors. However, the surrogates’ amounts were not taken into account in the above calculations of (2) and (3) since they were very small in amounts.

In addition, X-ray absorption near edge structure (XANES) was attempted to clarify bonding and coordination circumstances of Sr^2+^ and Cs^+^ incorporated into SC-GP samples. For Cs^+^, however, it was technically unsuccessful at the moment, due to the duplication of the L-edge of cesium with the K-edge of titanium in X-ray spectra.

## 3. Results and Discussion

### 3.1. Characteristics of PS-Ashes

Chemical compositions of PS-ashes are represented in Table 1. OTo3 has a usual composition. However, the others have unusual compositions. The high content of Cl in OTs2 would be attributed to refuse derived fuel (RDF), which contains much chlorine and moisture, while the high content of MgO in N45 to recycled plastic fuel (RPF), which contains much talc, commonly used as a filler in plastic appliances. Both RDF and RPF are used widely in Japan as boiler fuels, together with oils.

**Figure 1:**
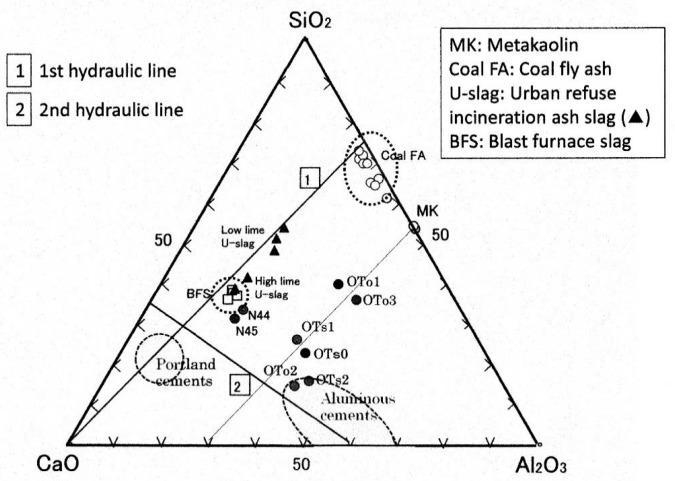
Ternary diagram plots of PS-ash fillers and other fillers studied so far.

To clearly grasp the features of the PS-ashes, the chemical compositions are plotted on the ternary diagram, as shown in Figure 1, in terms of CaO-Al_2_O_3_-SiO_2_ mass ratio. The plots encompass a wide range of PS-ashes. OTo/OTs series are plotted alongside with a trend line parallel to the first hydraulic line running through to the metakaolin point. On the other hand, N-series showed different features, plotted in the vicinity of blast furnace slag (BFS), a member of the first hydraulic line. The former group (OTo/OTs series) can be called high-alumina PS-ash, while the latter group (N-series) can be called low-alumina PS-ash. From another point of view, the PS-ashes can be classified into two groups—Ca-rich and Ca-poor. OTo1 and OTo3 are in the latter group, but the others are in the former group. Readers are kindly requested to refer to our past publication for relevant PS-ash compositions [31].

**Figure 2:**
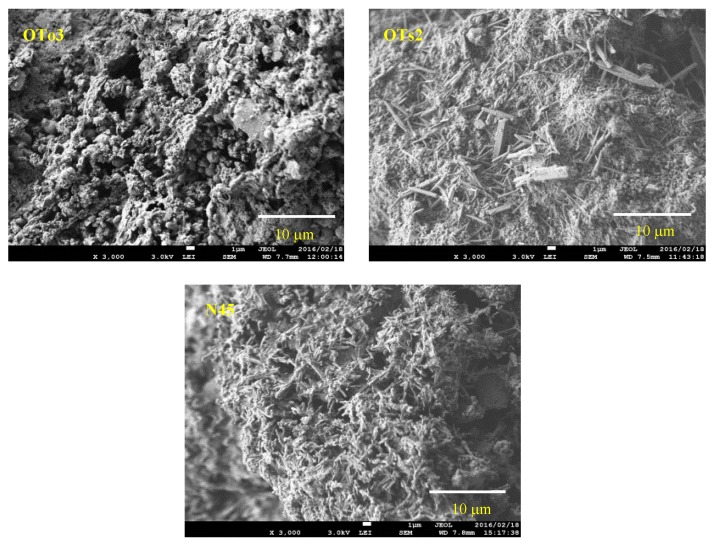
SEM images of the raw PS-ashes.

SEM images of the PS-ash are represented in Figure 2. Sponge-like porous textures peculiar to gel materials [33] can be seen for all the samples—most clearly for OTo3. Moreover, ettringite prevailed in OTo2 and N45 in acicular to prismatic forms. Particularly in OTs2, some ettringite crystals developed in stout prismatic form.

**Figure 3:**
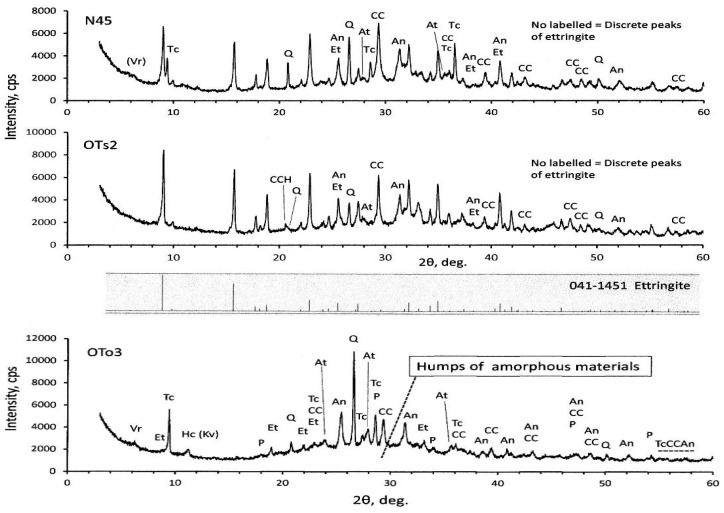
XRD diagram of raw OTo3. Keys refer to Table 3 and Table 5.

XRD diagrams of the PS-ash are represented in Figure 3. Calcite, quartz, and anhydrite were identified, irrespective of the PS-ash. In addition, humps are recognized around 2θ = 20°–40°. Particularly, the humps are very clear in OTo3, suggesting the presence of amorphous materials. OTo3 is characterized by less presence of ettringite, while the others (OTs2 and N45) show the pronounced presence of ettringite due to high SO_3_ contents, in addition to high LOI and moisture contents, as seen in Table 1.

It is important to classify the identified minerals on the basis of primary and secondary origins, as classified in Table 3. In OTo3, relict portlandite was detected, indicating that primarily quick lime (CaO) formed in the boiler. Water is generally sprinkled over the ash to prevent the ash from scattering after incineration, or sometimes the ash is pelletized. Accordingly, the quick lime slakes into portlandite and finally turns to calcite, in some cases to vaterite [31], due to reaction with CO_2_ in air. The ashes derived from plants and woods generally comprise K_2_O, CaO, and SiO_2_ as main components, in addition to MgO, P_2_O_5_, SO_3_, and Na_2_O as subordinate components [34]. Quartz may generate during the incineration in boilers. Anhydrite is a product of desulfurization. Generally, fluidized bed-type boilers are fed by limestone together with fuel coal for desulfurization, but for paper sludge incinerations, limestone is not required, since paper sludge contains much CaO. Moreover, calcite is usually used as filler and coating materials for ordinary grade paper. Contrary, kaolin or kaolin/calcite mixtures are used for high grade paper. Waste papers are so often fed to boilers as fuel, which also act as moisture absorber of wet paper sludge, so the origin of the Al_2_O_3_ component would be the kaolin. Strictly speaking, the presence of talc is because talc is so widely used as filler materials for paper and plastic appliances, so it remains in the ash of paper sludge, waste paper, and RPF combustions. A small amount of anorthite was identified, of which formation was also reported in literature for the ash from a pressurized fluidized bed-type boiler [16]. Vermiculite and hydrocalumite are secondary minerals formed during the water sprinkling. The presence of kovdorskite is also suspected other than hydrocalumite, judging from the 2θ positions. OTs2 and N45 show nearly the same XRD patterns—in which ettringite is prevailed—but talc is lacking in OTs2. It should be noted that hydrocalcite was identified in OTs2, of which the main peak is slightly different from the quartz peak, as indicated in Figure 3. Anorthite is also identified in minor quantities in both OTs2 and N45. Forsterite might be a primary origin, as discussed later in Section 3.3.

It should be stressed that raw PS-ashes are not completely amorphous, but abundant in crystalline phases—quite different from conventional geopolymer fillers such as metakaolin, water quenched blast furnace slag, coal fly ash, and so on.

### 3.2. Physical Properties of PS-Ash-Based Geopolymer

As shown in Table 4, for the #0 and #1 liquors, adequate values of L/F of PS-ash based geopolymer were from 1 to 1.5, depending on filler characters, particularly on specific surface areas and porous textures. Incidentally, a standard L/F is 0.4 for ordinary geopolymers in pastes [35]. OTs2 showed extremely slow setting with L/F 1.0, taking overnight, presumably due to the presence of so much chlorine, in addition to low specific surface area, so the specimen preparation for OTs2 was skipped in the present experiment. Other PS-ash-based geopolymer mixtures set within 40 min. Retarding effects of chlorine are recently confirmed by the experiment of using NaCl (Ichimiya, K., 2016, personal communication).

**Figure 4:**
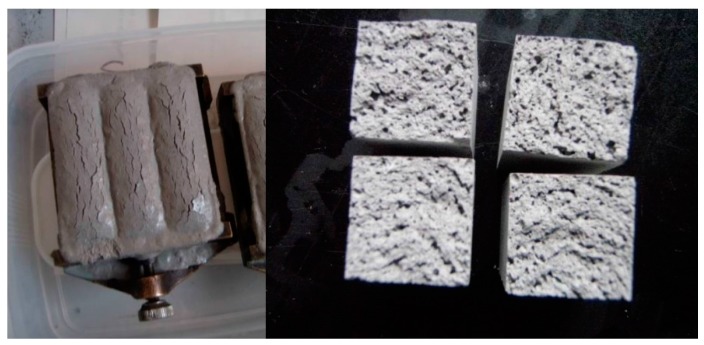
Swelling of hardened specimens and cross section of 0-1 specimen derived from OTo3 filler and #0 liquor.

It should be noted that when the #0 liquor was applied, the OTo3-based geopolymer mixtures swelled. Eventually, many pores of foaming origin were observed in the hardened specimens (Figure 4), resulting in smaller bulk densities, as indicated in Table 4. Presumably, free Al metal is included in this kind of PS-ash. The origin of the Al metal would be Al-laminated appliances (such as food containers), which might slip into the RDF used in the boiler plant of OTo3. However, at this moment, the Al metal could not be observed, even under the high power of scanning electron microscopy and energy dispersive spectroscopy (SEM-EDS), which is probably disseminated at the nanometer scale in the ash. Hydrogen gas is generated due to the high pH circumstances of the #0 liquor. To the contrary, no swelling was observed for the #1 liquor without mixing the caustic soda solution. Incidentally, this type of swelling is also reported for an RDF-ash [36] and hydrogen generation is confirmed by gas chromatography (Goda, H., 2012, oral communication).

Flexural strength of this lightweight material was relatively high, compared to the other non-swelled materials. It is postulated that nitride ions suppress the development of strength to a certain degree, due to the pH-lowering action of alkaline liquors [31,35]. However, in this study, this phenomenon was restricted to the specimens mixed with the #1 liquor.

### 3.3. XRD Results of Hardened Geopolymer

**Figure 5:**
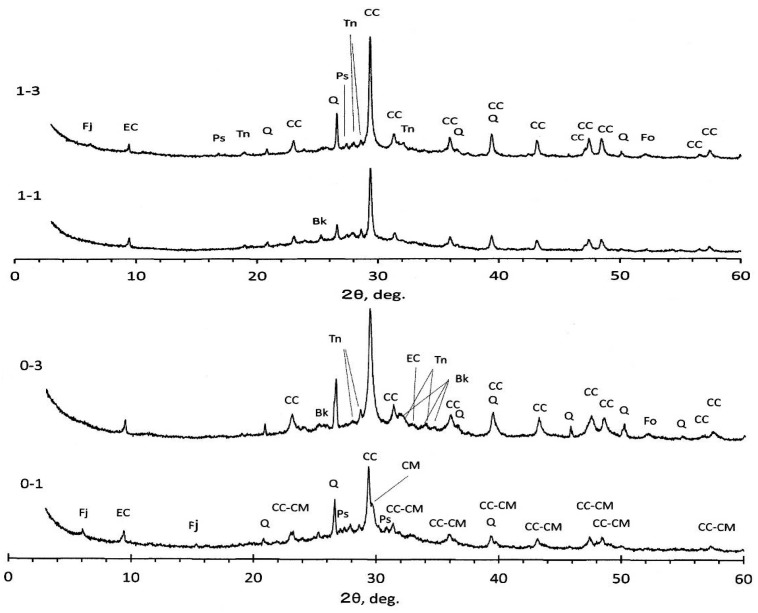
XRD diagrams of hardened bodies. Specimen keys refer to Table 4, and mineral keys refer to Table 3 and Table 5.

For the plain specimens, XRD diagrams are represented in Figure 5 for the #0 and #1 liquors, respectively. Quartz and calcite remained more or less intact, irrespective of sample species. However, judging from the variation of peak heights, some part of these minerals were reacted and diminished in highly alkaline circumstances, even for quartz. Quartz decrease was also observed in the literature [5]. Ettringite and anhydrite were not detected any more, and neither were anorthite and vermuculite. Instead, the formation of carbonate ettringite was observed, irrespective of specimen species. It is noted that magnesian calcite, (Ca, Mg)CO_3_, was identified for 0-1 specimen, of which peaks are located just a little bit higher than the 2θ angles of calcite, CaCO_3_, as shoulders. Peak patterns of magnesian calcite are quite different from dolomite, Ca, Mg(CO_3_)_2_. Formation of faujasite, a member of zeolite family, was observed for 0-1 and 1-3 specimens. Other identified minerals are pirssonite, burkeite, and thenerdite, depending on specimens—all formed accompanied with the geopolymerization of mixed alkaline liquors.

It is more plausible that the forsterite identified for specimens 0-3 and 1-3 might be a primary origin formed in the boiler. This mineral peaks might be concealed with anhydrite peaks in raw PS-ashes, and appeared intact according to the disappearance of anhydrite. Other than the crystalline phases mentioned above, there are amorphous phases more or less depending on specimens as so far postulated, judging from some humps around 2θ = 30°. For SC-GP specimens, no marked differences from plain specimens were observed, probably due to lower-level dosages of the surrogates. However, according to literature using metakaolin filler under higher level presence of nitrates, the formation of sodalite and cancrinite was reported as nitrate-bearing phases in addition to zeolite A and zeolite X, depending on NaOH concentrations [37].

Talking again about the faujasite, naturally-occurring faujasite generally has the chemical formula (Na_2_, Ca, Mg)_3.5_(Al_7_Si_17_O_48_)·32H_2_O, in which the S/N (SiO_2_/Na_2_O) molar ratio is 4.86. Incidentally, according to literature, naturally occurring faujasite has an S/N of around 4.5 [38]. It is well-known that synthetic faujasite is classified by S/N into zeolite X (lower than 3.0) and zeolite Y (higher than 3.0). XRD patterns represented in Figure 5 show that faujasite patterns are close to Na_2_Al_2_Si_2.4_O_8.8_·6.7H_2_O, where the S/N is 2.4, so the formation of zeolite X is more plausible, suggesting that the incorporation of Na-Al combination is large in the faujasite structure.

### 3.4. Reaction Process of PS-Ash-Based Geopolymers

Resultant minerals are summarized in Table 5, classified into three categories. The first is the intrinsic minerals of primary origin formed in boilers. The second is the extrinsic minerals of secondary origin formed due to the water sprinkling. The third is relevant to the geopolymerization of alkaline liquors with the minerals included in the PS-ashes. 

Referring to the hardening process of PS-ashes, the primary and the secondary minerals react with the alkaline liquors so that the recombination takes place, and minerals peculiar to the geopolymerization form. Thereby, forsterite may remain intact, while quartz and calcite may remain partially intact. Other minerals were completely consumed to yield a group of minerals called GP-minerals in Table 5, which are carbonate ettringite, faujasite, pirssonite, burkeite, and thenerdite, and sometimes magnesian calcite, other than amorphous gels which are truly matrices. The species of GP-minerals are dependent on the PS-ashes and alkaline liquors used. It is noteworthy to refer to the amorphous phases peculiar to geopolymers, which are currently believed to consist of two distinct phases—so called C-A-S-H gels and N-A-S-H gels—where C, A, S, H, and N denote CaO, Al_2_O_3_, SiO_2_, H_2_O, and Na_2_O, respectively. It is considered that in Ca-rich geopolymer systems, C-A-S-H is stable, while N-A-S-H is unstable, due to the occurrence of an ion exchange of Ca^2+^ for Na^+^. Accordingly, N-A-S-H turns gradually to C-A-S-H and eventually diminishes [39,40,41,42]. Strictly speaking, initial C-A-S-H and N-A-S-H may finally become (Ca, N)-A-S-H gels after the ion exchange.

### 3.5. Immobiization Ratios of Sr^2+^ and Cs^+^

Results of the 4 + 2 week age samples are tabulated in Table 6, together with relevant data for calculations. High immobilization ratios of Sr^2+^ and Cs^+^ were reached for 0-1SC and 1-1SC, both using OTo3 as filler, whereas Cs^+^ immobilization was relatively low for 0-3SC and 1-3SC, both using N45 as filler. It should be noted that specimen 1-3SC showed over-scale, indicating poor immobilization of Sr^2+^, so more detailed measurements of ICP were skipped for this specimen. Cs^+^ will be mentioned later. 

Regarding the immobilization mechanisms of Sr^2+^ and Cs^+^, two possibilities are considered. One is that these ions are incorporated into faujasite and the other is into geopolymer gels (GP-gels). Faujasite would accommodate both Sr^2+^ and Cs^+^, since it consists of sodalite cages with openings of 0.74 nm in diameter. Incidentally, according to Shannon and Prewitt [43], ionic radii of Sr^2+^ and Cs^+^ are 0.118–0.144 nm and 0.167–0.188 nm, respectively, depending on coordination numbers from 6-hold to 12-hold. Gobbinsite, (Na,Ca_0.5_)_6_(Al_6_Si_10_)O_32_·12H_2_O—a zeolite family mineral—was also identified as a candidate mineral in a previous study [31]. However, judging from XRD peak intensities, the formation of faujasite is in very small quantities, and the formation of GP-gels is prevalent due to the polycondensation of applied alkaline liquors. 

It is postulated that there are two kinds of GP-gels—C-A-S-H and N-A-S-H [39,40,41,42]. N-A-S-H is considered to be unstable under the co-presence of C-A-S-H, due to the ion exchange of Ca^2+^ for Na^+^. Sr^2+^ may be preferentially incorporated into C-A-S-H. We considered at the moment that the high MgO content of specimens 1-3SC (Table 1) may interfere the stable formation of C-A-S-H in young ages, so 1-3SC showed over-scale for Sr^2+^. Incidentally, the stability and instability of GP-gels are also called “a complex multistep reaction process”, leading to a phase-mixed product in the case of N-A-S-H [44]. That is, a non-equilibrium-to-equilibrium transition of GP-gels. However, little has been studied in the case of C-A-S-H.

For Cs^+^ incorporation, N-A-S-H and faujasite may play an important role. However, it is predictive that N-A-S-H becomes unstable with elapsed time. Therefore, the immobilization ratio of Cs^+^ may decrease with the ion exchange, resulting in some Cs^+^ depletion. In the long run, however, with the formation of new C-A-S-H phase, (C, N)-A-S-H, Cs^+^ may be again incorporated. In order to confirm this hypothesis, immobilization ratios of prolonged ages were measured up to 24 weeks, nearly 6 months. The results are shown in Table 7.

For Sr^2+^, higher immobilization ratios were generally maintained, irrespective of material age. However, 1-3SC showed unstable immobilization ratios. Strangely, we found that it turned dramatically up to 91.73% at 12 week of age, but it turned down again at 24 weeks of age, suggesting unstable incorporation of Sr^2+^ in this mixture. As mentioned, the high MgO content may be a cause of this instability, and there also may be some transitional stages in the evolution of C-A-S-H gels with elapsed time. 

For Cs^+^, decreasing immobilization ratios were found at 12 weeks of age, as expected, and this phenomenon is extremely remarkable for the #0 liquor. However, these ratios again rose up at 24 weeks of age, as expected.

As a whole, 1-1SC applying #1 liquor exhibited stable immobilization ratios, irrespective of material ages. To prevent the fluctuation of immobilization ratios, one of the measures would be high-temperature curing, which accelerates the polycondensation reactions to yield stable GP-gels in a short period of time. Incidentally, high immobilization ratios were also reported for Cs^+^ incorporated into alkali-activated fly ash matrices cured at 85 °C and 120 °C, reaching 98.56%–99.78% immobilization ratios that were analyzed by toxic characteristic leaching procedure (TCLP), using the pH 2.88 leaching solution of glacial acetic acid [35]. Retentions of 95.2% and 81.4% are reported, respectively, for Sr^2+^ and Cs^+^ in 0.01 M NaOH solution, using magnetic zeolite nanocomposites [45]. 

Finally, we tentatively tried to convert the hardened SC-GP into slags to reduce volumes. Fusion temperature and duration were 1400 °C-1 h for 0-1SC and 1-1SC, and 1300 °C-1 h for 0-3SC and 1-3SC, respectively. Moistures of GP-liquor origin as well as evaporates represented as LOI in Table 1 were taken into account in calculations. Results were tabulated at the right-bottom corner of Table 6, showing high immobilization ratios for both Sr^2+^ and Cs^+^. Therefore, slag conversion would be one of the options for eternal burials as proposed in a previous study [31]. 

### 3.6. XANES

**Figure 6:**
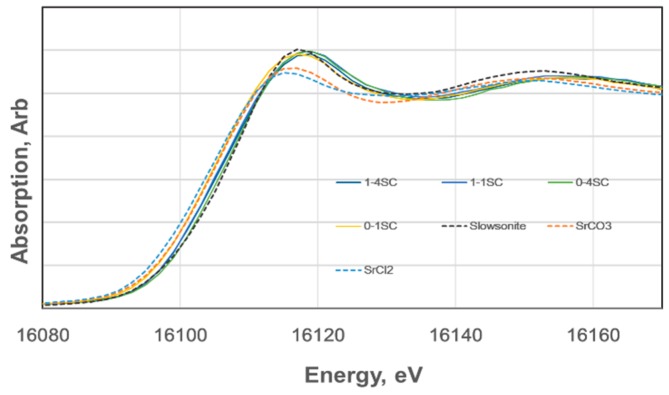
X-ray absorption near edge structure (XANES) spectra of hardened bodies for strontium K-edge.

XANES spectra of the K absorption edge of strontium for SC-GP specimens of 4 + 2 week age were compared with the spectra of the standard samples of SrCl_2_, SrCO_3_, and slawsonite, (Sr, Ca)Al_2_Si_2_O_8_). The former two are reagents, and slawsonite is a naturally-occurring mineral involving strontium in the structure from Sarusaka, Kochi, Japan. As shown in Figure 6, spectra are relatively well-separated from each other for the standard samples, indicating that the chemical states of Sr^2+^ were different among SrCl_2_, SrCO_3_, and slawsonite. On the other hand, XANES spectra of strontium in the SC-GP specimens are almost in a bundle, indicating that chemical states of Sr^2+^ in these specimens are nearly identical. The obtained results indicate that the chemical states of Sr^2+^ in SC-GP specimens resemble that in slawsonite. Incidentally, in slawsonite, Sr^2+^ is surrounded by O^2−^ in 7-hold coordination with a mean M-O distance of 0.263 nm [46]. Deducting the ionic radius of O^2−^, which is 0.126 nm, after Shannon and Prewitt [43], 0.137 nm is obtained as the net ionic radius of Sr^2+^. However, this ionic radius of Sr^2+^ corresponds to 8-hold coordination, according to [47].

It should be stressed again that 1-3SC showed less capability of Sr^2+^ at 4 + 2 and 24 weeks of age. However, it was found that the immobilization ratio became enhanced to 91.73% at the intermediate age of 12 weeks. XANES measurements were performed by using 4 + 2 week age specimens, but there is about one month time lag for the actual measurements, due to time sharing schedules of the facility. Therefore, it is difficult, at the moment, to tell the spectra of 1-3SC, whether coming from embryo stage of C-A-S-H gels due to slow coagulation or coming from well-developed and matured C-A-S-H or (C, N)-A-S-H gels. According to literature [48,49], Sr^2+^ incorporations are unstable in metakaolin-based geopolymers, in which N-A-S-H gels form preferentially. In other words, depletion of Sr^2+^ takes place to yield SrCO_3_ in air, but no depletion of Cs^+^ occurs, meaning 100% incorporation of Cs^+^ into N-A-S-H. Formation of Sr(OH)_2_ and as SrCO_3_ is also reported in the metakaolin-based geopolymer, where the clinoptilolite zeolite encapsulating Sr^2+^ and Cs^+^ is mixed [50].

At the moment, no spectral profiles corresponding to SrCO_3_ were observed in the XANES spectra, even for 1-3SC, which showed highly fluctuating immobilization ratios of Sr^2+^ with elapsed time, as mentioned. Therefore, we concluded that no depletion of Sr^2+^ took place in our SC-GP specimens and that the fluctuations may be attributed to the transitional GP-gel structures of C-A-S-H or (C, N)-A-S-H. That is, for instance, differences in degrees of polymerization and/or differences in tetrahedral order–disorder configurations, which would be sensitive to acidic conditions of the leaching test. The XANES technique will become a powerful measure to clarify the bonding and coordination circumstances of matrix gels in geopolymers.

## 4. Conclusions

Paper mill wastes—PS-ashes—can be solidified at ambient temperature by geopolymerization technique, by mixing with alkaline liquors designated as #0 liquor (having an aqueous Na-metasillicate composition) or #1 liquor (having aqueous Na-disilicate composition), independently. It has been found that PS-ashes consist of a high quantity of crystalline phases, accompanied with some amorphous phases such that they can be regarded as semi-crystalline fillers, quite different from the conventional fillers of metakaolin, glassy blast furnace slag, and glassy coal fly ash, which consist of almost entirely amorphous materials.

Primarily, solidification and strength tests were carried out, preparing solidified paste specimens, plain, and SC-GP-containing non-radioactive Sr(NO_3_)_2_ and CsNO_3_ as surrogates. Specimens were cured at 20 °C-100% RH for 4 weeks. Subsequently, they were air-dried up to 24 weeks of age. Finally, immobilization ratios of Sr^2+^ and Cs^+^ were determined for the SC-GP specimens at 6, 12 and 24 weeks, respectively, by leaching test with pH 4.01 buffer solution. The following conclusions are summarized.

(1) Constituent minerals of the PS-ashes are classified into two groups: the primary or intrinsic minerals formed in boilers, and the secondary or extrinsic minerals due to water sprinkling. The primary and secondary minerals decompose into so-called GP-minerals during the geopolymerization process.
(2) Hazardous waters contaminated with radioisotopes can be solidified by geopolymer technique, making use of the porous textures of PS-ashes, which can absorb much water. The leaching test generally showed high immobilization ratios—98%–99% and 97%–98% for Sr^2+^ and Cs^+^, respectively, depending on material ages, alkaline liquors, and filler species. However, some specimens showed highly fluctuating immobilization ratios for both of Sr^2+^ and Cs^+^ with elapsed time.
(3) XANES spectra of strontium K-edge of SC-GP specimens were measured at 6 weeks of age, and were discussed in comparison with the slawsonite spectrum. The obtained results showed that chemical environments of Sr^2+^ incorporated into the PS-ash based GP-gels are similar to slawsonite.

## Figures and Tables

**Table 1:** Physicochemical characteristics of paper sludge (PS)-ashes used as geopolymer filler, mass %.

	SiO_2_	TiO_2_	Al_2_O_3_	Fe_2_O_3_	MnO	CaO	MgO	Na_2_O	K_2_O	P_2_O_5_	SO_3_	Cl	Others ^1^	Total (%)
OTo3	30.94	1.55	37.37	1.88	0.03	18.57	3.58	0.33	0.81	1.56	2.71	0.41	0.28	100.02
OTs2	10.39	2.07	28.47	2.19	0.04	27.50	1.61	1.47	3.83	5.31	5.88	10.89	0.36	100.01
N45	21.87	0.77	13.75	2.58	0.37	34.95	10.44	0.80	0.78	3.56	9.25	0.42	0.45	99.99
	Apparent density, g/cm^3^					Specific surface area, cm^2^/g, Blaine								
OTo3	2.50					6460								
OTs2	1.87					1980								
N45	2.26					5680								

^1^ Other minor elements: ZnO, CuO, BaO, SrO, NiO, PbO, ZrO_2_, CeO_2_, Cr_2_O_3_, Bi_2_O_3_, etc. SrO contents: 0.023%, 0.029%, and 0.066% for OTo3, OTs2, and N45, respectively. Cs_2_O was not detected within detection limits. LOI, loss on ignition at 1000 °C: OTo3/6.00 (1.66)%, OTs2/32.57 (23.42)%, N45/22.10 (12.49)%, respectively, other than the total. In parentheses H_2_O(-) values determined at 105 °C are indicated.

**Table 2:** Compositions of plain geopolymer pastes, mass %.

Liquor	L/F ^1^	Filler	Na-Silicates ^2^	Water	Total (%)
#0	1.50	40.00	14.13	45.87	100
	1.20	45.45	12.85	41.70	100
#1	1.50	40.00	14.55	45.45	100
	1.20	45.45	13.23	41.32	100

^1^ L/F = Liquor (Dry Na-silicates + water)/Filler mass ratio; ^2^ Dry Na-silicates, Na_2_O·SiO_2_ or Na_2_O·2SiO_2_.

**Table 3:** Summary of XRD results of PS-ashes.

PS-ash Minerls			PS-ash			Remark
Key	Minerals of primary origin		OTo3	OTo2	N44	In boilers
Q	Quartz	SiO_2_	++++	++	+++	Liberated SiO_2_
An	Anhydrite	CaSO_4_	+++	++	++	Sulfur capture
At	Anorthite	CaAl_2_Si_2_O_8_	++	+	+	Recombination
Fo	Forsterite	Mg_2_SiO_4_	-	-	(+)	Recombination
Tc	Talc	Mg_3_(OH)_2_Si_4_O_10_	+++	-	++	RPF-origin
	Amorphous		++	+	+	Recombination
Key	Minerals of secondary origin					After sprinkling
CC	Calcite	CaCO_3_	++	+++	+++	CO_2_ in air
CCH	Hydrocalcite	CaCO_3_·H_2_O	-	+	-	CO_2_ in air
Po	Portlandite	Ca(OH)_2_	+	-	-	Slaked only
Et	Ettringite	Ca_6_Al_2_(SO_4_)_3_(OH)_12_·26H_2_O	+	++++	+++	Recombination
Hc	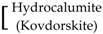	Ca_2_Al(OH)_6_Cl·2H_2_O	+			Recombination
(Kv)		Mg_2_(PO_4_)(OH)·3H_2_O	(+)	-	-	Recombination
Vr	Vermiculite	Mg_3_Si_4_O_10_(OH)_2_	+	-	(+)	Recombination

++++ strong; +++ medium; ++ small; + very small; (+) presence suspected; - undetected. RPF: recycled plastic fuel.

**Table 4:** Resultant flexural strength and bulk density of PS-ash-based geopolymers.

Filler	Key ^1^	L/F	Flexural Strength, 4 wk ^2^	Bulk Density (g/cm^3^)		
		#0 Liquor	(MPa)	4 wk (D_0_)	4 + 2 wk (D_1_)	12, 24 wk (D_2_, D_3_) ^3^
OTo3	0-1	1.5	1.19	1.29	0.85	0.82
	0-1SC	1.5	1.22	1.29	0.84	0.82
N45	0-3	1.2	0.82	1.50	1.10	0.98
	0-3SC	1.2	0.99	1.49	1.08	0.97
		**#1 liquor**				
OTo3	1-1	1.5	1.19	1.56	1.04	1.02
	1-1SC	1.5	1.07	1.58	1.05	1.02
N45	1-3	1.2	1.51	1.52	1.05	0.97
	1-3SC	1.2	1.19	1.54	1.05	0.98

^1^ SC: Sr(NO_3_)_2_ and CsNO_3_ added as surrogate, and no SC indicating Plain. 0-2, 0-2SC, 1-2, 1-2SC of OTo2 with L/F 1.0 were not indicated here, since hardened bodies were not prepared. Refer to the text; ^2^ wk: weeks; ^3^ After 12 weeks, no more marked change in bulk density was observed.

**Table 5:** Resultant -minerals in PS-ash based geopolymers in comparison with PS-ash minerals and whole reaction process.

	PS-ash Minerals					Minerals in PS-ash Based Geopolymers		
Key	Minerals of primary origin		Remark		Key			Remark
Q	Quartz	SiO_2_	Partially intact	➡		Quartz	SiO_2_	
An	Anhydrite	CaSO_4_	Consumed					
At	Anorthite	CaAl_2_Si_2_O_8_	Consumed					
Fo	Forsterite	Mg_2_SiO_4_	Remain intact	➡		Forsterite	Mg_2_SiO_4_	
Tc	Talc	Mg_3_(OH)_2_Si_4_O_10_	Consumd					
	Amorphous		Recombination					
Key	Minerals of secondary origin							
CC	Calcite	CaCO_3_	Partially intact	➡		Calcite	CaCO_3_	
CCH	Monohydrocalcite	CaCO_3_·H_2_O	Consumed		CM	Magnesian calcite	(Ca, Mg)CO_3_	Insoluble
Po	Portlandite	Ca(OH)_2_	Consumed		EC	Carbonate-ettringite ^2^		Insoluble
Et	Ettringite ^1^		Consumed		Fj	Faujasite ^3^		Insoluble
HC	Hydrocalumite	Ca_2_Al(OH)_6_Cl·2H_2_O	Consumed		Ps	Pirssonite	Na_2_Ca(CO_3_)_2_·2H_2_O	Sparingly soluble
(Kv)^4^	(Kovdorskite)	Mg_2_(PO_4_)(OH)·3H_2_O	(Consumed)		Bk	Burkeite	Na_6_(CO_3_)(SO_4_)_2_	Sparingly soluble
Vr	Vermiculite	Mg_3_Si_4_O_10_(OH)_2_	Consumed		Tn	Thenerdite	Na_2_SO_4_	Soluble
	Alkaline liquor	Na_2_O-SiO_2_-H_2_O		➡		Amorphous	C-A-S-H, N-A-S-H	

^1^ Ca_6_Al_2_(SO_4_)_3_(OH)_12_·26H_2_O; ^2^ Ca_6_Al_2_(CO_3_)_3_(OH)_12_·26H_2_O; ^3^ (Na_2_, Ca, Mg)_3.5_(Al_7_Si_17_O_48_)·32H_2_O; ^4^ Presence suspected.

**Table 6:** Resultant immobilization ratios of strontium and cesium for 4 + 2 week age specimens with some relevant data.

	%Filler	To 12.5 g	Surrogates,	Sr^2+^	Cs^+^	ICP, 421 nm	ICP, 459 nm
		Sample (g)	as Nitrate (mg)	(mg)	(mg)	Sr^2+^ (ppb)	Cs^+^ (ppb)
#0 liquor							
0-1SC	61.43	7.68	76.8	31.8	52.4	530	11,580
0-3SC	62.74	7.84	78.4	32.5	53.5	450	121,800
#1 liquor							
1-1SC	60.19	7.52	75.2	31.1	51.3	4400	14,040
1-3SC	66.67	8.33	83.3	34.5	56.8	Scale over	131,500
	To 125 g leaching solution		Immobilization ratio SC-GP			Immobilization ratio SC-GP-Slag	
	Sr^2+^ (μg)	Cs^+^( μg)	Sr^2+^ (%)	Cs^+^ (%)		Sr^2+^ (%)	Cs^+^ (%)
#0 liquor						#0 liquor	
0-1SC	66.25	1447.5	99.79	97.24		98.38	97.38
0-3SC	56.25	15,225.0	99.83	71.54		93.19	97.36
#1 liquor						#1 liquor	
1-1SC	550	1755.0	98.23	96.58		97.60	100
1-3SC	-	16,437.5	-	71.06		97.11	99.48

ICP: Induction Coupled Plasma Spectroscopy. SC-GP: geopolymer including Sr^2+^ and Cs^+^.

**Table 7:** Summary of immobilization ratios as a function of material age.

	Sr^2+^	4 + 2 wk	12 wk	24 wk	Cs^+^	4 + 2 wk	12 wk	24 wk
0-1SC	#0 liquor	99.79	99.80	99.89	#0 liquor	97.24	Over-scale	97.48
0-3SC		99.83	99.83	99.88		71.54	Over-scale	98.30
1-1SC	#1 liquor	98.23	98.27	98.59	#1 liquor	96.58	90.95	98.77
1-3SC		Over-scale	91.73	Over-scale		71.06	46.16	95.65
